# Comparative Clinical Outcomes Between Successive Generations of Liberty Trifocal Intraocular Lenses

**DOI:** 10.3390/jcm15176926

**Published:** 2026-09-07

**Authors:** Juan J. Prados-Carmona, Mayelín Pérez-Perdomo, Álvaro Sánchez-Ventosa, Marta Villalba-González, Miguel González-Andrades, Alberto Villarrubia-Cuadrado, Antonio Cano-Ortiz, Yolanda Jiménez-Gómez

**Affiliations:** 1 Department of Ophthalmology, Maimonides Biomedical Research Institute of Cordoba (IMIBIC), Reina Sofia University Hospital—University of Cordoba, 14004 Cordoba, Spain; 2 Department of Anterior Segment, Cornea and Refractive Surgery, Hospital Arruzafa, 14012 Cordoba, Spain; 3 Departamento de Ciencias de la Salud y Biomédicas, Universidad Loyola Andalucía, 41704 Sevilla, Spain; 4 Faculty of Biomedical Sciences and Sports, European University of Andalucía, 29010 Málaga, Spain

**Keywords:** cataract surgery, intraocular lens, visual and refractive outcomes, patient-reported satisfaction, spectacle independence

## Abstract

**Purpose:** To compare the clinical and functional outcomes of two successive generations of a trifocal intraocular lens (IOL), the original Liberty 677MY and the optimized Liberty 677CMY. To the best of our knowledge, this is the first head-to-head comparison between two successive generations of any IOL model reported. **Methods:** This retrospective study included 42 patients (84 eyes) who underwent bilateral phacoemulsification with implantation of either the Liberty 677MY (26 eyes) or the Liberty 677CMY (58 eyes). The primary outcome was postoperative binocular intermediate visual acuity (VA). Secondary outcomes included monocular intermediate VA, monocular and binocular distance and near VA, the proportion of patients or eyes achieving ≤0.1 LogMAR, and defocus curves. Exploratory outcomes included spectacle independence, patient-reported satisfaction (Quality of vision—QoV- and Catquest-9SF), photic phenomena, and intraoperative or postoperative complications. **Results:** At 3 months, binocular UIVA differed by approximately 0.10 LogMAR in favor of the 677CMY cohort, which was considered clinically significant, although the difference did not reach statistical significance (*p* = 0.052). Supporting this tendency, monocular UIVA was significantly better with the 677CMY IOL (*p* = 0.012). Also, a significantly higher percentage of patients and eyes achieved binocular and monocular UIVA ≤ 0.1 LogMAR with the 677CMY IOL (*p* = 0.025). Defocus curves additionally showed significant between-group differences at intermediate defocus levels (−1.00 and −1.50 D; *p* < 0.05), favoring the 677CMY cohort. No between-group differences were observed for any distance VA parameter. Binocular near VA and near-vision thresholds were also comparable between groups, although a greater proportion of eyes achieved monocular UNVA ≤ 0.1 LogMAR with the 677CMY IOL (*p* = 0.016). Spectacle use, overall satisfaction, photic phenomena profile, and postoperative complications were comparable between groups. **Conclusions:** The optimized Liberty 677CMY exhibited a favorable pattern of intermediate visual performance compared with the 677MY while maintaining comparable distance and near VA and a similar safety profile. These findings suggest potential clinical benefits of the design modifications, although larger prospective studies are warranted to confirm these observations.

## 1. Introduction

**Key points:**

- What is already known: Trifocal IOLs provide functional vision across distances, though the first-generation Liberty 677MY showed relatively limited intermediate performance.
- What this study adds: The newer Liberty 677CMY showed a favorable pattern of intermediate visual performance, while maintaining comparable near and distance vision, safety, and spectacle independence.
- Implications: Comparing successive IOL generations helps identify potential clinical improvements and supports evidence-based lens selection in cataract surgery.

Cataract surgery is one of the most frequently performed surgical procedures in ophthalmology and has evolved into a refractive intervention that not only restores visual transparency but also offers the potential for spectacle independence [1]. Advances in phacoemulsification techniques and the development of intraocular lenses (IOLs) have been key drivers of this transformation [2].

Multifocal IOLs emerged as an alternative to traditional monofocal lenses, allowing functional vision at multiple distances [3]. Initially, bifocal lenses were implanted, providing improvements in both near and distance vision but with limitations at intermediate distances. Subsequently, the introduction of trifocal IOLs marked a paradigm shift by improving intermediate vision without compromising distance and near performance [4], thereby enhancing the overall functional visual performance and providing higher levels of spectacle independence [5], ultimately resulting in a substantial improvement in patients’ quality of life [6]. Consequently, trifocal IOLs have become a well-established premium option for refractive cataract surgery and presbyopia correction [4]. A wide variety of trifocal IOL models are now available, and the specific optical properties of each model may affect its suitability according to individual patient features and visual demands [7]. For this reason, studies aimed at characterizing the optical performance of each IOL are necessary to establish the optimal clinical indications for these implants.

In recent years, the Liberty Bi-Flex 677MY trifocal IOL (Medicontur Medical Engineering Ltd., Zsámbék, Hungary) has been the subject of several clinical studies. Early pilot studies demonstrated that patients implanted with this IOL exhibited adequate visual recovery, supporting its safety and efficacy [8,9]. Subsequent clinical studies confirmed its refractive stability and spectacle independence [10,11] whereas other studies reported high levels of patient satisfaction [12]. Overall, the Liberty 677MY IOL represented an effective and well-tolerated option for presbyopia correction, even when evaluated in its toric version [13]. However, a deeper understanding of the performance of any IOL model requires comparative analyses, which are essential to assess its relative performance and suitability. Indeed, a clinical trial has demonstrated that the 677MY design improves intermediate and near visual acuity, while achieving higher rates of spectacle independence compared to standard monofocal IOLs [3]. Other comparative studies showed that the Liberty 677MY delivers favorable distance and near visual performance compared with other trifocal IOLs such as the AT LISA tri 839M, with reported higher rates of 20/20 uncorrected distance visual acuity (UDVA) and uncorrected near visual acuity (UNVA) (56% vs. 41% and 83% vs. 66%, respectively) [14]. Another study further suggested that the Liberty 677MY provides a favorable defocus curve profile for distance and near vergences compared with other trifocal IOLs (i.e., AT LISA tri 839M, AcrySof IQ, and PanOptix) [15]. However, the 677MY IOL appeared to provide inferior intermediate visual performance compared with some other trifocal IOLs, such as the FineVision POD F, AT LISA tri 839M, AcrySof IQ, or PanOptix [15,16] which could represent a limitation of this IOL design when considering intermediate visual demands. Beyond visual acuity (VA) outcomes, a previous study has reported other relevant findings for the Liberty 677MY, including a high proportion of eyes within ± 0.50 D of the refractive target, favorable contrast sensitivity outcomes, and a low rate of capsulotomy compared with other reported trifocal platforms [14].

Currently, an optimized version of this lens, named Liberty 677CMY, has been developed to improve intermediate vision while maintaining the excellent distance and near visual outcomes of the original model. Both IOLs share the same hydrophilic acrylic material, but the 677CMY incorporates refinements in its optical design intended to optimize light distribution. To date, only one published study has reported clinical outcomes with this new Liberty IOL. In that prospective study published by our group, the Liberty 677CMY achieved high levels of uncorrected VA across all distances, with 100% of patients achieving ≥20/25 for distance, 92% for intermediate, and 80% for near vision [17].

The literature includes numerous comparisons between different models or designs—such as bifocal vs. trifocal or refractive vs. diffractive—that have expanded our understanding of emerging technologies [18]. However, no studies have directly compared successive versions of the same lens model, making it difficult to determine whether technical refinements translate into meaningful clinical benefits. The Liberty IOL provides a particularly relevant case since, despite the extensive body of evidence available for the original 677MY model, it has not yet been established whether the new 677CMY version delivers optimized outcomes. To our knowledge, the present study is the first comparative study evaluating two consecutively marketed versions of the same IOL—Liberty 677MY and 677CMY—, thereby providing a direct clinical comparison of the successive design iterations. We aimed to assess whether the optimized IOL was associated with improved intermediate visual performance compared with the original model.

## 2. Materials and Methods

### 2.1. Study Design and Participants

An observational, retrospective study was conducted at Arruzafa Hospital (Córdoba, Spain) to compare the two consecutively marketed versions of the same IOL. All patients were treated as part of routine clinical practice. The inclusion and exclusion criteria for the recruitment of the patients were as follows:

- Inclusion criteria: patients older than 50 years with an indication for cataract surgery and/or a desire for spectacle independence.
- Exclusion criteria: corneal topographic astigmatism greater than 1.0 D or irregular astigmatism, previous ocular trauma or previous ocular surgery (including refractive procedures), and the presence of active ocular diseases such as retinal pathologies, glaucoma, corneal dystrophies, or clinically significant dry eye. Patients with anatomical abnormalities such as optic nerve atrophy, iris atrophy, or zonular weakness were also excluded, as well as those receiving systemic or topical treatments with the potential to affect visual performance. Finally, individuals with uncontrolled systemic diseases that could increase surgical risk or bias visual outcomes, such as poorly controlled diabetes, were also excluded.

A total of 42 patients were included. All participants underwent bilateral implantation with either the Liberty 677MY [first generation, *n* = 13 patients (26 eyes)] or the Liberty 677CMY [second generation, *n* = 29 patients (58 eyes)]. The two cohorts were recruited sequentially, reflecting the clinical introduction of the two IOL generations. Once the newer-generation Liberty 677CMY became clinically available, implantation of the first-generation IOL was discontinued. Follow-up assessments were conducted at 3 months for visual outcomes and at 18 months for postoperative adverse effects. While previous publications by our group reported the outcomes of each cohort independently [8,17] the present study was the first to present a head-to-head comparison of the two.

This study was conducted in accordance with the tenets of the Declaration of Helsinki. All experimental protocols were approved by the Ethics Committee of Arruzafa Hospital (Córdoba, Spain), and written informed consent was obtained from all participants.

### 2.2. Technical Characteristics of the Liberty IOLs

Although exact CE-marking and commercialization dates are not publicly available, available manufacturer documentation and the chronology of published clinical studies suggest that the Liberty 677MY was introduced around 2016–2017, whereas the optimized Liberty 677CMY became available approximately in 2022–2023.

The Liberty Bi-Flex 677MY and 677CMY IOLs (Medicontur) are single-piece, aspheric, biconvex IOLs with an overall diameter of 13.0 mm and a 6.0-mm optic. Both models feature an asymmetric, non-angulated haptic design with posterior vaulting, which provides enhanced stability within the capsular bag. They are manufactured from a hydrophilic acrylate/methacrylate copolymer similar to that used in other trifocal IOLs [14]. The optical design is based on a diffractive-refractive platform with a centralized 3.0-mm diffractive zone containing seven diffractive rings with progressive apodization (the height of the diffractive steps gradually decreases from the center toward the periphery), thereby aiming to minimize light scatter. The Liberty design concentrates the diffractive rings within the central optic, while the peripheral optic remains predominantly refractive. The optical architecture provides a primary distance focus together with additional focal points of +1.75 D for intermediate vision and +3.50 D for near vision, corresponding to viewing distances of approximately 60–67 cm and 40 cm, respectively. The newer Liberty 677CMY model incorporates an optimized optical design; however, detailed information regarding the specific optical modifications has not been publicly disclosed. Both IOL models were available in powers ranging from +8.0 to +35.0 D in 0.5 D increments. Regarding their differences, the 677MY model requires manual loading, while the 677CMY is supplied preloaded, thereby simplifying the surgical procedure. The lenses implanted in both cohorts corresponded to the spherical version, without a toric component for astigmatism correction. The present comparative analysis was restricted to spherical models to maintain homogeneity between cohorts and specifically evaluate the impact of the optical modifications introduced in the 677CMY design.

### 2.3. Surgical Procedure

All surgeries were performed using a standardized approach to maximize comparability of results between cohorts. All interventions in both cohorts were performed by the same three experienced surgeons using the standard phacoemulsification technique. Under topical anesthesia, a 2.2-mm corneal incision was made, followed by the creation of a manual continuous curvilinear capsulorrhexis of approximately 5.0 mm and subsequent phacoemulsification of the crystalline lens. The Liberty IOLs, either the 677MY or the 677CMY, were implanted in the capsular bag using a generic injector for the 677MY model or the preloaded injector provided with the 677CMY model. The lens power was calculated with the Barrett Universal II formula using an A constant of 118.9, targeting emmetropia as the refractive endpoint. At the conclusion of surgery, a postoperative regimen consisting of a combination of antibiotics and corticosteroids was prescribed following standardized protocols.

### 2.4. Data Acquisition and Variables

After signing the informed consent form, all patients underwent a comprehensive ophthalmologic examination before and after the surgical procedure. A case report form was used to collect the following baseline data:

- Demographic variables: age and sex.
- Clinical variables: surgical indication (cataract surgery or clear lens extraction—CLE), cataract maturity grade assessed by slit-lamp biomicroscopy, and intraocular pressure (IOP) measured using Goldmann applanation tonometry. Keratometry and the corneal aberrometric profile were analyzed using corneal topography (Pentacam; Oculus Optikgeräte GmbH, Wetzlar, Germany), whereas axial length and other biometric parameters required for IOL power calculation were obtained via optical biometry (IOLMaster 500; Carl Zeiss Meditec, Jena, Germany). Additional examinations included an endothelial cell count, anterior segment optical coherence tomography (AS-OCT), and a fundus examination to exclude any concomitant ocular pathology.

At 3 months post-intervention, the following data were collected:

- Primary outcome: binocular intermediate VA, measured at 66 cm and assessed under uncorrected and distance-corrected conditions (UIVA and DCIVA). VA was measured using standardized optotypes and expressed on the LogMAR scale.
- Secondary outcomes: monocular UIVA and DCIVA at 66 cm; monocular and binocular uncorrected and distance-corrected distance VA (UDVA and CDVA) at 4 m; monocular and binocular uncorrected and distance-corrected near VA (UNVA and DCNVA) at 40 cm; the proportion of patients and eyes achieving a VA of ≤0.1 LogMAR; and binocular defocus curves (ranging from +2.00 to −4.00 D in 0.50 D vergence steps).
- Exploratory outcomes: manifest refraction (spherical equivalent) and patient-reported outcomes (PROs). The latter comprised vision-related quality of life, patient satisfaction, spectacle independence, difficulty performing daily activities, and perception of photic phenomena. Subjective PROs were assessed using the Quality of Vision (QoV) questionnaire developed by the research group [17] and the validated Catquest-9SF questionnaire [19]. Responses obtained with the Catquest-9SF questionnaire were transformed into Rasch-calibrated scores using a 4-Andrich rating scale model for statistical purposes [19].

Finally, intraoperative and postoperative complications were also evaluated. For long-term complications, a minimum individual follow-up period of 18 months was established; therefore, only patients meeting this criterion were considered for this analysis.

**Figure 1:**
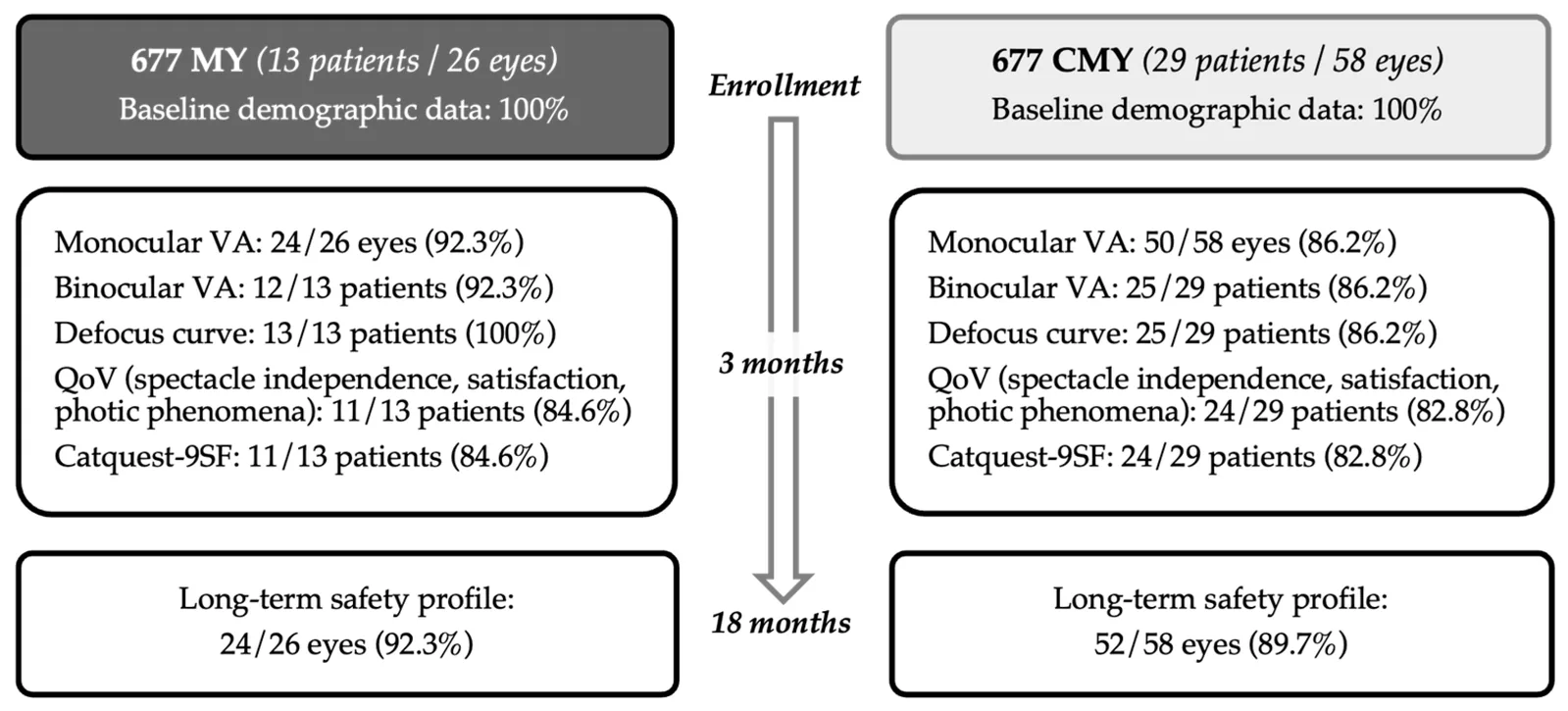
Patient- and eye-level data availability across outcome assessments. The diagram shows the number of patients and eyes contributing to each outcome at 3 and 18 months.

Figure 1 summarizes the availability of patient- and eye-level data for each outcome at the 3- and 18-month follow-up assessments, indicating the number of patients and eyes contributing to each analysis.

### 2.5. Statistical Analysis

Statistical analysis was performed using SPSS software version 25.0 (SPSS Inc., Chicago, IL, USA). Sample size calculation for the comparison of two independent means of postoperative binocular intermediate VA between cohorts indicated that 12 patients/group would be required to detect clinically relevant mean differences of 0.1 LogMAR [20], assuming an SD of 0.08 LogMAR, a two-sided α level of 0.05 and 80% statistical power, with a 10% allowance for potential losses to follow-up. The sample size calculation was performed at the patient level using binocular intermediate VA as the reference outcome; the other visual-acuity measures were considered additional clinically relevant secondary outcomes and were not used for the original sample size determination.

Quantitative variables were expressed as mean ± standard deviation (SD) and/or median (interquartile range [IQR]), depending on the distribution, whereas categorical variables were expressed as absolute frequency distributions and percentages. Statistical analyses were conducted separately at the patient and eye levels, as appropriate for each variable.

(a) Patient-level analysis. Normality of data distributions was assessed using the Shapiro–Wilk test. Continuous variables were compared between cohorts using the independent-samples Student’s *t*-test for Gaussian data, and the Mann–Whitney U test for non-normally distributed data. VA across defocus levels was analyzed using repeated-measures ANOVA, with sphericity assessed using Mauchly’s test and the Huynh-Feldt correction applied when the assumption of sphericity was violated; post hoc pairwise comparisons were adjusted using the Bonferroni method for multiple comparisons. Categorical variables were analyzed using Fisher’s exact test for binary outcomes. Ordinal variables were compared using exact two-sided Mann–Whitney U tests and Bonferroni correction was applied within predefined families of related outcomes according to their questionnaire domain to control for Type I error.
(b) Eye-level analysis. For monocular outcomes, both eyes of a patient were considered correlated observations because both eyes received the same IOL type. Continuous eye-level outcomes were analyzed using linear mixed-effects models, with IOL type as a fixed effect and patient identification as a random effect to account for the correlation between fellow eyes. Eye was specified as the repeated within-patient measurement. Model assumptions were assessed based on the model residuals using histograms, Q–Q plots, the Shapiro–Wilk test, and residuals-versus-fitted values plots. Deviations from normality were interpreted in conjunction with graphical assessment. Extreme observations were reviewed for data accuracy and were retained when confirmed as valid measurements. For eye-level outcomes that showed deviations from normality, a sensitivity analysis was performed using generalized estimating equations (GEE) with a normal distribution and identity link. Categorical eye-level outcomes were analyzed using GEE with a binomial distribution and logit link for binary outcomes and an ordinal logit model for ordinal outcomes. For all GEE models, patient identification was specified as the subject variable, eye as the within-subject variable, and an exchangeable working correlation structure with a robust covariance estimator was used.

A two-sided *p*-value < 0.05 was considered statistically significant.

## 3. Results

### 3.1. Study Population

Baseline demographic characteristics of the patients in the two studied cohorts are summarized in Table 1.

At the patient level, no statistically significant between-group differences were observed in age and gender (*p* > 0.05). At the eye level, the proportion of eyes undergoing cataract surgery versus CLE was 38.5% in the 677MY cohort, and 62.1% of cases in the 677CMY cohort (OR, 2.6; 95% CI, 0.7–10.0; *p* = 0.161). Among eyes undergoing cataract surgery, the median preoperative cataract grade was 3 (IQR: 1–4) in those who received the 677MY IOL, and 2 (IQR: 1–4) in those implanted with the 677CMY IOL (OR, 0.4; 95% CI, 0.07 to 2.4; *p* = 0.309). Regarding IOP, axial length, corneal power (mean keratometry), and manifest refraction (spherical equivalent), no statistically significant differences were observed between the two cohorts (*p* > 0.05). No additional ophthalmic comorbidities or clinically relevant abnormalities were detected in ancillary tests (i.e., endothelial cell count or AS-OCT) among the enrolled patients.

All surgeries were performed using a standardized approach to maximize comparability of results between cohorts. In the 677MY cohort, the lens power calculated by biometry was 22.9 ± 3.2 D, and the final implanted lens power was 22.9 ± 3.3 D, whereas both values were 22.4 ± 3.2 D in the 677CMY cohort. The calculated and implanted lens powers were identical in 82 of 84 eyes (97.6%), while the remaining two eyes showed a difference of 0.5 D. All surgical procedures were completed without intraoperative complications.

### 3.2. Postoperative Refractive and Visual Acuity Outcomes

**Figure 2:**
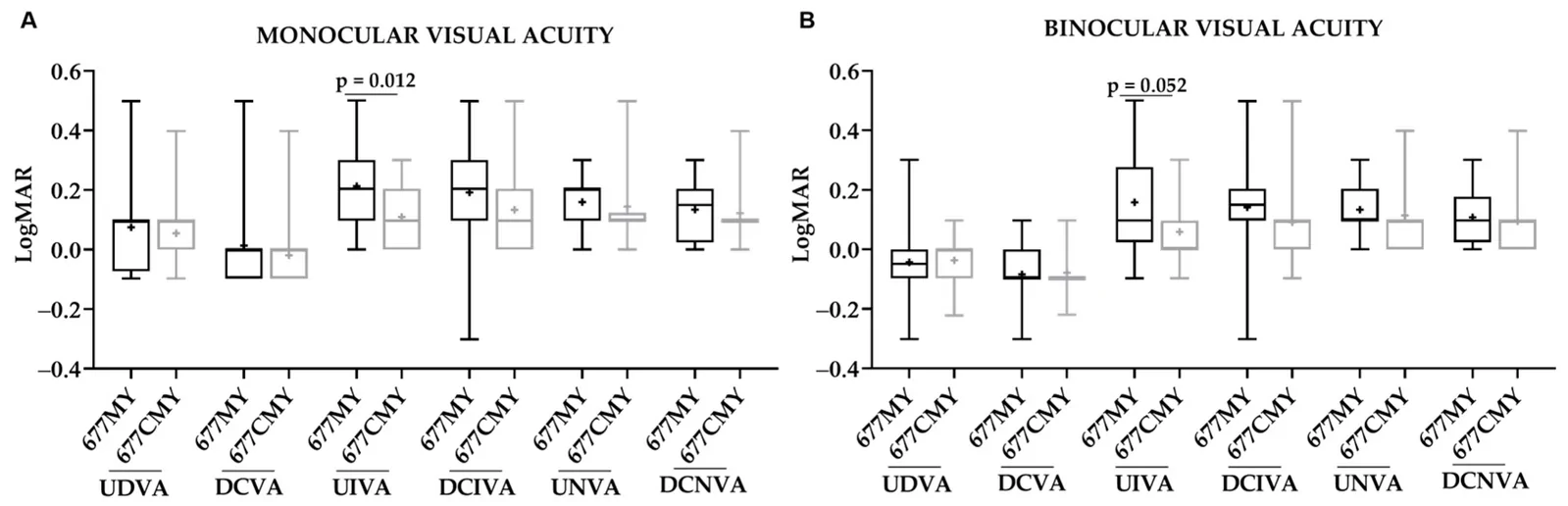
Postoperative visual acuity at the 3-month follow-up. (**A**) Monocular visual acuity (677MY: 24 eyes from 12 patients; 677CMY: 50 eyes from 25 patients). (**B**) Binocular visual acuity (677MY: 12 patients; 677CMY: 25 patients). Box-and-whisker plots show the median and interquartile range (IQR), with whiskers extending from the minimum to the maximum value; the mean is indicated by a plus sign (+). Monocular visual acuity was compared using a linear mixed-effects model, whereas binocular visual acuity was analyzed using the Mann–Whitney U test. VA: visual acuity; UDVA: uncorrected distance visual acuity; CDVA: corrected distance visual acuity; UIVA: uncorrected intermediate visual acuity; DCIVA: distance-corrected intermediate visual acuity; UNVA: uncorrected near visual acuity; DCNVA: distance-corrected near visual acuity.

**Figure 3:**
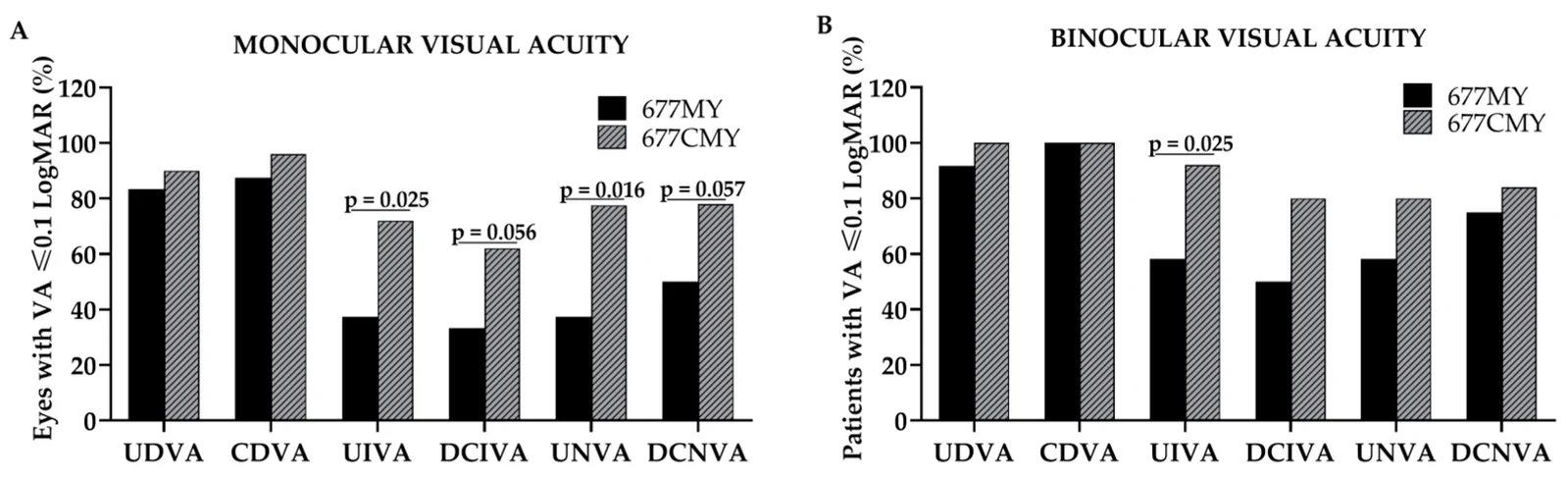
Percentage of eyes and patients achieving visual acuity ≤ 0.1 LogMAR at the 3-month follow-up. (**A**) Monocular visual acuity (677MY: 24 eyes from 12 patients; 677CMY: 50 eyes from 25 patients). (**B**) Binocular visual acuity (677MY: 12 patients; 677CMY: 25 patients). Monocular data were compared using generalized estimating equations, whereas binocular data were analyzed using Fisher’s exact test. VA: visual acuity; UDVA: uncorrected distance visual acuity; CDVA: corrected distance visual acuity; UIVA: uncorrected intermediate visual acuity; DCIVA: distance-corrected intermediate visual acuity; UNVA: uncorrected near visual acuity; DCNVA: distance-corrected near visual acuity.

By the 3-month follow-up, spherical equivalent (SE) was comparable between the 677MY and 677CMY cohorts, with means of +0.18 ± 0.33 D and +0.12 ± 0.42 D, respectively (estimated mean difference [EMD], −0.018; 95% CI, −0.20 to 0.16; *p* = 0.842). In addition, no statistically significant differences between the cohorts were found in distance vision (monocular and binocular UDVA and CDVA; *p* > 0.05) (Figure 2A,B). Likewise, a similar proportion of eyes and patients achieved distance VA of ≤0.1 LogMAR (*p* > 0.05; Figure 3A,B). For the primary outcome, binocular UIVA showed a numerical advantage in the 677CMY, with a median (IQR) of 0 (0–0.01) LogMAR compared with 0.10 (0.02–0.30) LogMAR in the 677MY cohort; however, the between-group difference did not reach statistical significance (*p* = 0.052; Figure 2B). Among secondary intermediate-vision outcomes, a higher proportion of patients achieved binocular UIVA ≤ 0.1 LogMAR with the 677CMY IOL (95% vs. 58%; OR, 8.2; 95% CI, 1.4 to 47.2; *p* = 0.025) (Figure 3B). Monocular UIVA was also better in the 677CMY cohort (mean ± SD, 0.11 ± 0.09 vs. 0.21 ± 0.16; EMD, −0.10 LogMAR; 95% CI, −0.20 to −0.02; *p* = 0.012) (Figure 2A), with a higher percentage of eyes achieving ≤0.1 LogMAR (72% vs. 37%; OR, 4.3; 95% CI, 1.2 to 15.1; *p* = 0.025) (Figure 3A). Regarding near vision, binocular VA and patient-level thresholds were also comparable between cohorts, although a higher proportion of eyes achieved monocular UNVA ≤ 0.1 LogMAR with the 677CMY IOL (76% vs. 37%; OR, 5.3; 95% CI, 1.4 to 20.3; *p* = 0.016) (Figure 3A).

**Figure 4:**
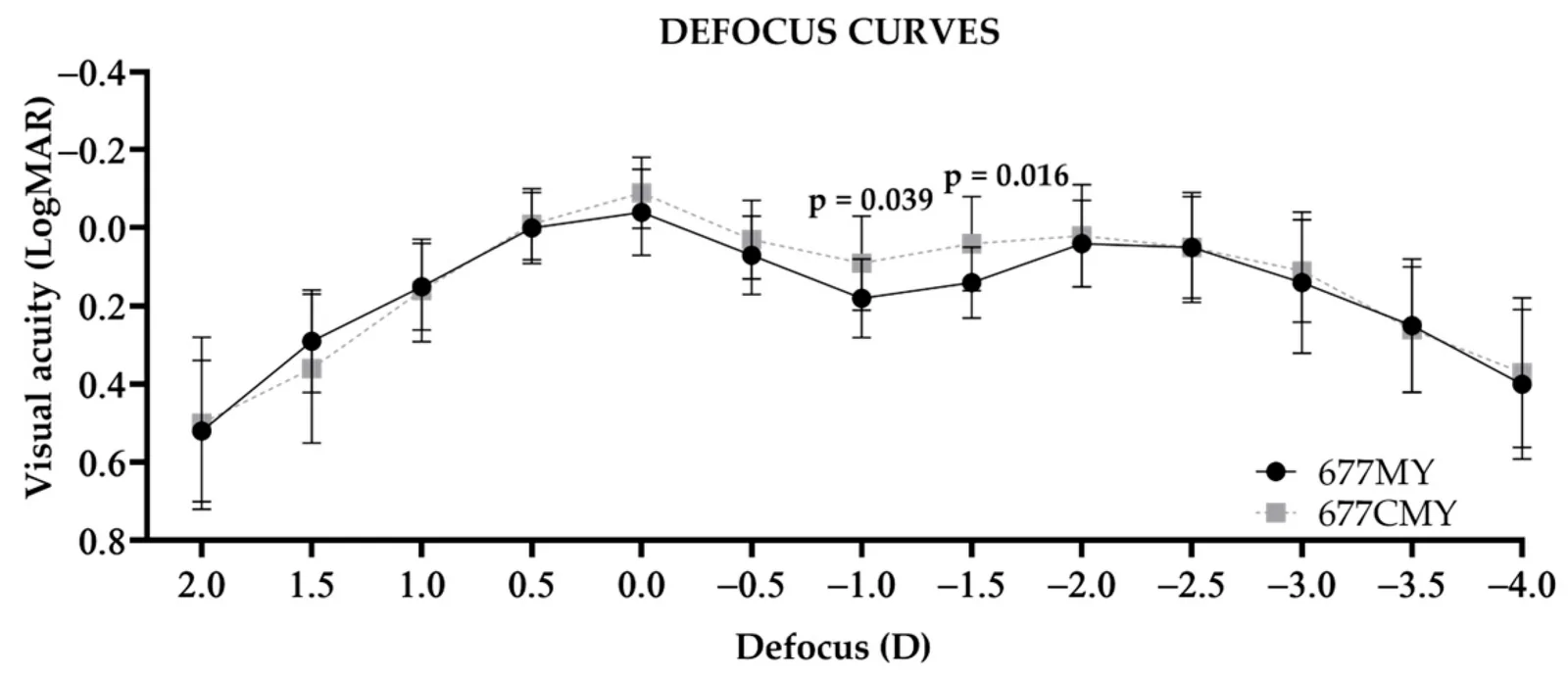
Binocular defocus curve values at the 3-month follow-up (*n* = 13 and *n* = 25 patients in the 677MY and 677CMY groups, respectively). Values are shown as mean ± SD. The data were analyzed using the repeated-measures ANOVA test. *p* < 0.05 (pairwise comparisons after Bonferroni adjustment).

Postoperative binocular defocus curves showed a characteristic trifocal profile in both cohorts, with peaks of best VA at 0 D (distance vision), −1.5 D (intermediate vision), and −3.0 D (near vision) (Figure 4). In terms of distance vision (0 D) and near vision (−3.0 D), results were equivalent between the two cohorts (*p* > 0.05). At intermediate defocus levels, the 677CMY cohort showed better VA than the 677MY cohort at −1.0 D (EMD, −0.09 LogMAR; 95% CI, −0.17 to −0.005; *p* = 0.039) and −1.5 D (EMD, −0.1 LogMAR; 95% CI, −0.17 to −0.02; *p* = 0.016). Although these pairwise differences were statistically significant after Bonferroni adjustment, the overall defocus-curve profile was not statistically significant (*p* = 0.393; Figure 4).

### 3.3. Patient-Reported Outcomes

**Figure 5:**
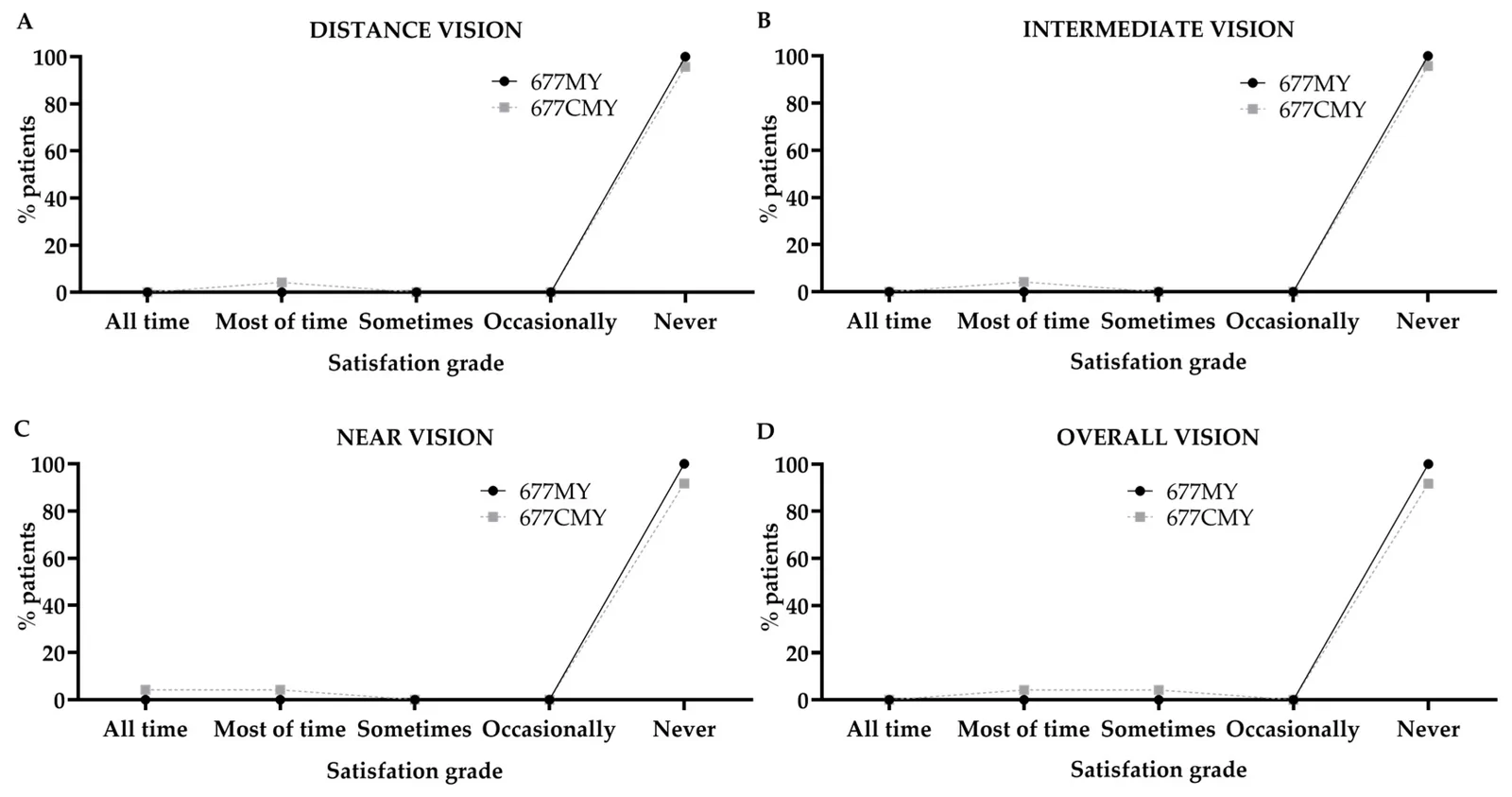
Frequency of spectacle use at multiple distances at the 3-month follow-up (*n* = 11 and *n* = 24 patients in the 677MY and 677CMY groups, respectively). (**A**) Distance vision; (**B**) intermediate vision; (**C**) near vision; (**D**) overall vision. Values represent the proportion of patients (%). Fisher’s exact test was used for categorical data, whereas the Mann–Whitney U test was used for ordinal data. Bonferroni correction was applied for multiple comparisons.

Our analysis of spectacle use frequency showed that the majority of patients implanted with the 677MY and 677CMY lenses reported never using spectacles at any of the evaluated distances. Comparative analysis between both cohorts revealed no statistically significant differences in spectacle use for distance, intermediate, near, or overall vision (*p* > 0.05; Figure 5A–D).

**Figure 6:**
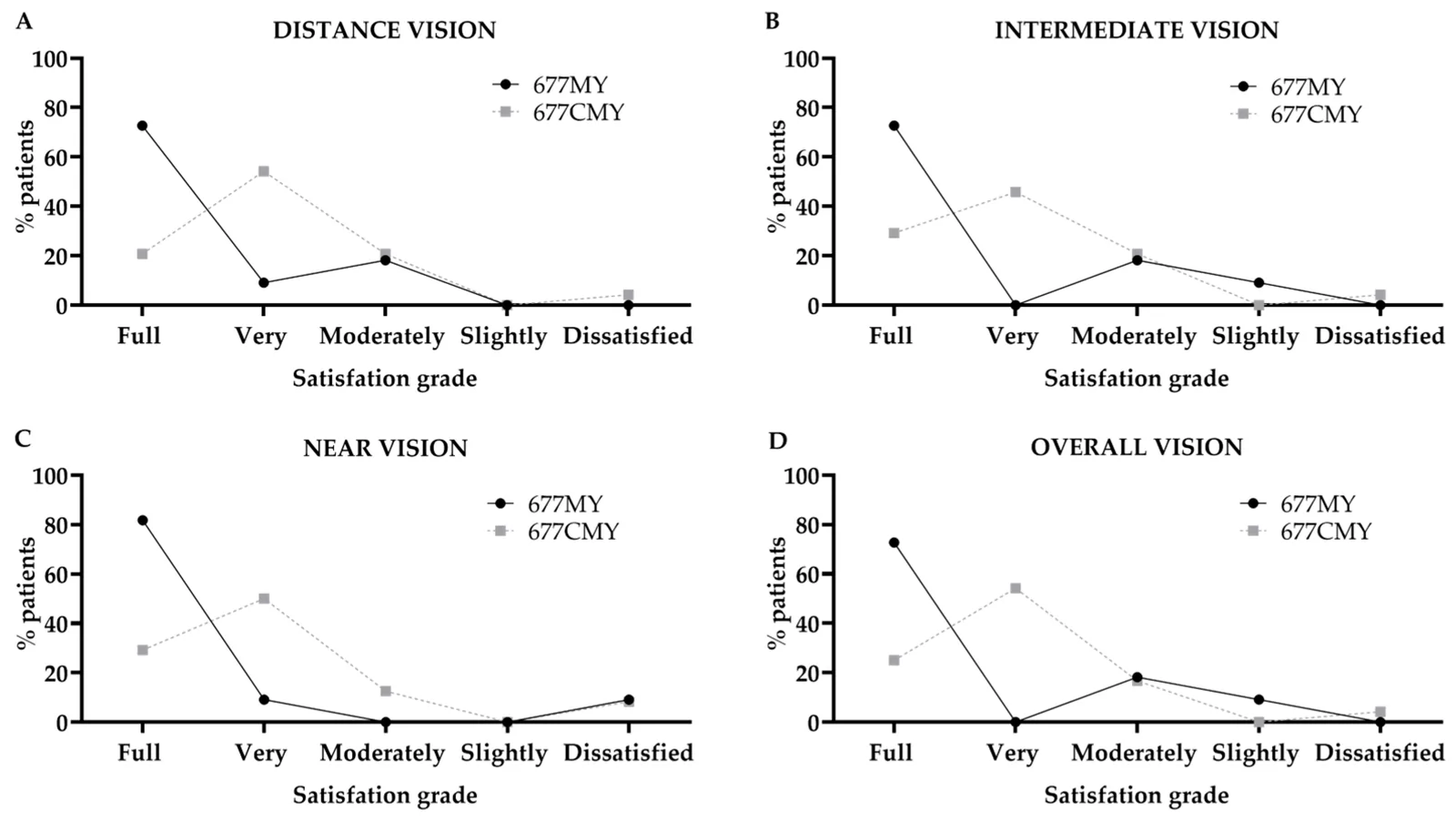
Postoperative visual satisfaction evaluated at multiple distances at the 3-month follow-up (*n* = 11 and *n* = 24 in the 677MY and 677CMY groups, respectively). (**A**) Distance vision; (**B**) intermediate vision; (**C**) near vision; (**D**) overall vision. Values represent the proportion of patients (%). Fisher’s exact test was used for categorical data, whereas the Mann–Whitney U test was used for ordinal data. Bonferroni correction was applied for multiple comparisons.

In terms of vision-related satisfaction, a high percentage of patients (>72%) reported being “fully or very satisfied” with the implanted IOL—either the 677MY or 677CMY—across all distances and with overall vision, with no statistically significant differences between the lenses. No statistically significant differences were observed between the two IOLs in patient satisfaction at any of the evaluated distances or for overall vision (*p* > 0.05; Figure 6A–D).

**Figure 7:**
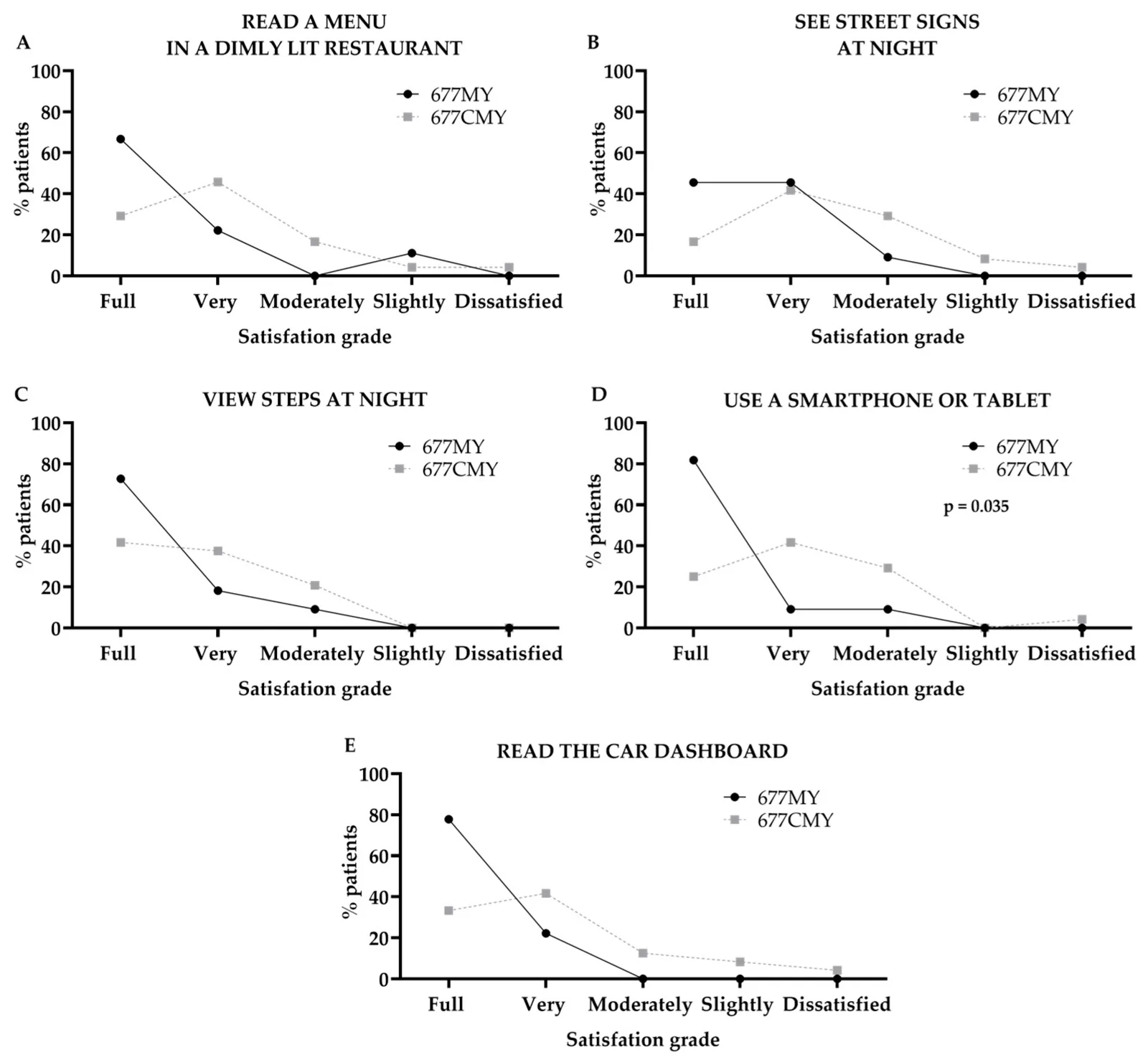
Distribution of patient responses regarding satisfaction levels when performing daily activities (*n* = 11 and *n* = 24 in the 677MY and 677CMY cohorts, respectively). (**A**) Read a menu in a dimly lit restaurant; (**B**) see street signs at night; (**C**) view steps at night; (**D**) use a smartphone or tablet; (**E**) read the car dashboard. Values represent the proportion of patients (%). Fisher’s exact test was used for categorical data, whereas the Mann–Whitney U test was used for ordinal data. Bonferroni correction was applied for multiple comparisons.

Subsequently, patients were also asked about their satisfaction level when performing daily activities. Overall, both IOL models provided high rates of responses indicating “full or high satisfaction” (ranging from 58.4% to 100% of patients), and no statistically significant differences were found (*p* > 0.05; Figure 7A–E). In addition, no statistically significant differences were observed between the distribution of responses from the 677MY and 677CMY cohorts for most activities, such as reading the car dashboard, identifying steps under low-light conditions, seeing traffic signs at night, and reading a menu in a dimly lit restaurant (*p* > 0.05; Figure 7A–C,E); however, our results revealed a statistically significant difference in the distribution of responses regarding visual satisfaction with the use of a smartphone or tablet between both cohorts (*p* = 0.035; Figure 7D), with the 677MY cohort showing a higher proportion of fully satisfied patients with respect to the 677CMY cohort. To further characterize these findings obtained from our questionnaire, we next administered the validated Catquest-9SF questionnaire. The Catquest-9SF questionnaire also includes items for evaluating overall visual difficulties in daily life, the degree of satisfaction with current vision, and visual difficulties when performing common daily tasks. No statistically significant differences were reported between the two cohorts (*p* > 0.05), showing high levels of self-reported visual function and satisfaction in daily activities in both cohorts.

**Figure 8:**
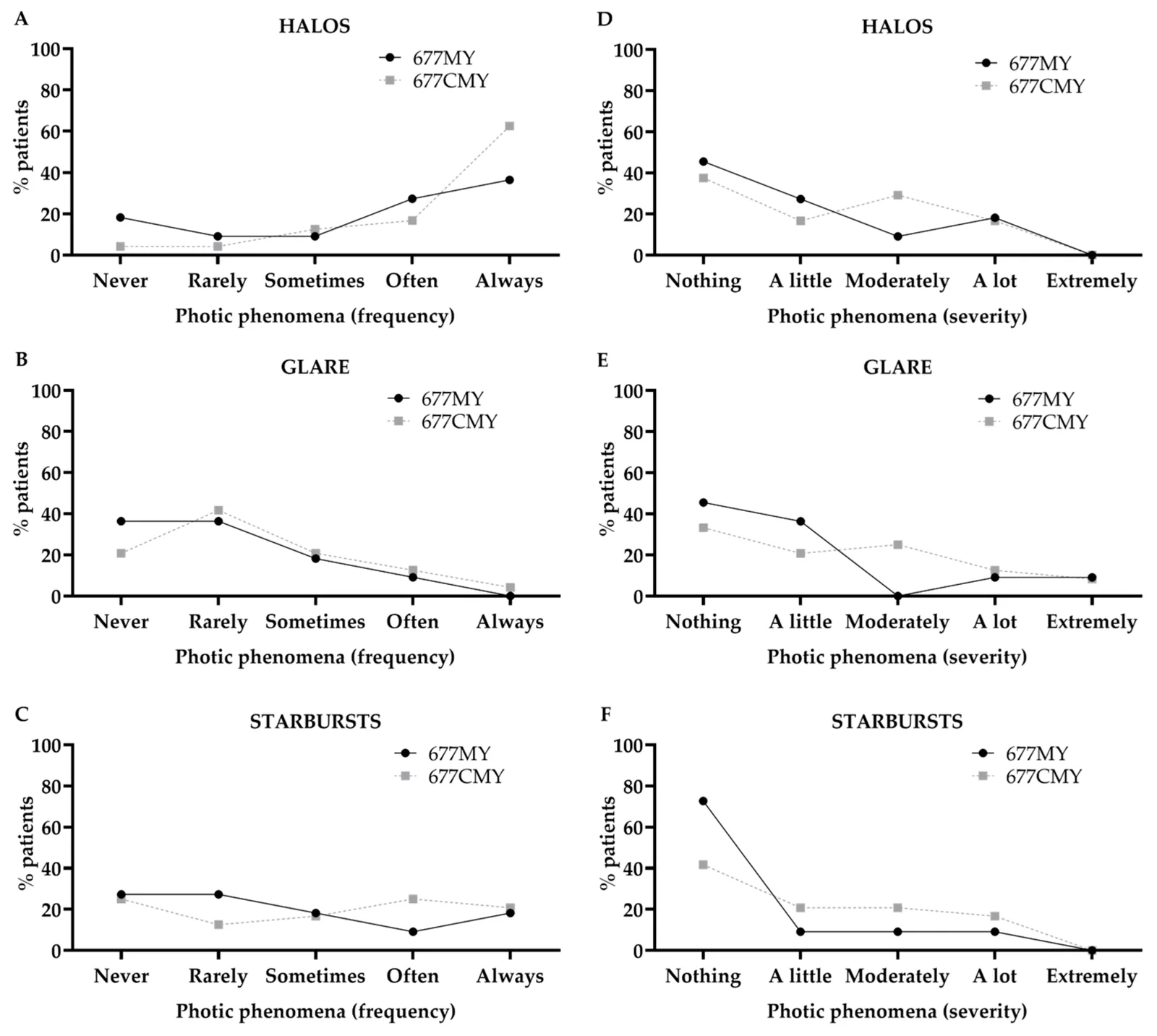
Distribution of the subjective data concerning the frequency and severity of photic phenomena (halos, glare and starburst) at the 3-month post-surgery follow-up (*n* = 11 and *n* = 24 in the 677MY and 677CMY groups, respectively). (**A**–**C**) Frequency of photic phenomena (halos, glare, and starbursts); (**D**–**F**) severity of photic phenomena (halos, glare, and starbursts). Values represent the proportion of patients (%). Fisher’s exact test was used for categorical data, whereas the Mann–Whitney U test was used for ordinal data. Bonferroni correction was applied for multiple comparisons.

Finally, our comparative analysis of photic phenomena—halos, starbursts, and glare—showed no statistically significant differences between cohorts in terms of either reported frequency or perceived bothersomeness (*p* > 0.05; Figure 8A–F). In both cohorts, most patients reported experiencing these phenomena but rated their impact as minimally disturbing (scores of 1–2 on a 5-point scale) (Figure 8A–F).

### 3.4. Long-Term Postoperative Complications

Long-term postoperative complications at 18 months among eyes in the two studied cohorts are detailed in Table 2. The overall rate of postoperative complications was 41.7% in the 677MY cohort and 26.9% in the 677CMY cohort, with no statistically significant difference between cohorts (OR = 0.5; 95% CI, 0.1 to 2.1; *p* = 0.358). Among the individual complications, Nd:YAG capsulotomy was the most frequent event, occurring in 41.7% of eyes implanted with the 677MY lens and 19.2% of eyes implanted with the 677CMY lens; this difference was not statistically significant after accounting for the correlation be-tween fellow eyes within the same patient (OR = 0.3; 95% CI 0.1 to 1.4; *p* = 0.142). Cystoid macular edema (CME) occurred in 3.7% of eyes in the 677CMY cohort and was not observed in the 677MY cohort, while refractive enhancement with laser-assisted in situ keratomileusis (LASIK) was performed in 3.7% of eyes in the 677CMY cohort and in none of the eyes in the 677MY cohort. Owing to the very low number of events for these individual complications, these outcomes were reported descriptively without inferential statistical comparisons. No additional postoperative complications were recorded.

## 4. Discussion

Trifocal IOLs are considered the premium standard in refractive cataract surgery [6]. Among these, the Liberty IOL has been extensively studied, with most published studies to date focusing on the first-generation Liberty 677MY [8,12,15,16]. These studies demonstrated reliable distance and near visual outcomes, although intermediate performance appeared to be relatively weaker compared with other trifocal models (e.g., AT LISA tri 839M, AcrySof IQ, and PanOptix) [12,15,16]. The Liberty 677CMY was developed as an optimized version designed to enhance intermediate vision while preserving satisfactory distance and near visual outcomes, safety profile, and patient satisfaction. Reports regarding this optimized model [17] suggested a more competitive visual performance, although direct head-to-head comparisons with the 677MY were lacking.

No statistically significant differences were observed between cohorts in terms of baseline demographic characteristics. However, the descriptive differences in surgical indication and cataract maturity should be considered when interpreting between-cohort comparisons. Notably, the absence of preoperative relevant corneal aberrations in both cohorts is of particular importance, as corneal higher-order aberrations and coma have been reported to adversely affect postoperative intermediate and near visual outcomes [9]. All surgeries were performed following a standardized procedure, and no intra-surgical complications, and IOL power calculation and implantation were highly consistent, with identical calculated and implanted IOL powers in 82 of 84 eyes (97.6%).

By the 3-month follow-up, spherical equivalent was comparable between the two Liberty IOL versions, with mean values close to emmetropia in both groups. As spherical equivalent can significantly affect postoperative intermediate vision [9], these findings suggest that the 677CMY potentially maintains similar refractive outcomes to the 677MY.

Our VA analysis revealed that, although both IOL versions provided excellent distance and near vision, the optical optimization incorporated into the 677CMY may have preferentially benefited intermediate visual performance. Although the primary outcome, binocular intermediate VA, did not reach statistical significance, the numerical difference between cohorts (0.10 LogMAR for UIVA) corresponded to approximately one line on a standard VA chart and may be considered clinically relevant for daily activities requiring intermediate vision, such as digital device use. Previous comparative IOL studies have used a 0.10 LogMAR margin as a reference for intermediate visual improvement [20]. Additionally, this numerical tendency was accompanied by a consistent pattern of significantly more favorable intermediate-vision secondary outcomes for the 677CMY, including a higher likelihood of achieving good binocular and monocular UIVA (≤0.1 LogMAR). Similarly, the defocus curves showed a more favorable intermediate vision performance with the 677CMY at selected defocus levels after adjustment for multiple comparisons. However, because these were secondary findings, they should be considered supportive rather than confirmatory evidence of a potential benefit of the 677CMY IOL. Taken together, these results suggest that the new 677CMY lens may provide favorable intermediate visual performance within the range reported by other trifocal IOLs, such as the AT LISA tri 839M and PanOptix [15].

Regarding patient-reported outcomes, both Liberty IOL versions achieved high rates of spectacle independence across all distances (≥90% of patients), with no significant between-cohort differences. These results are consistent with those reported by Cervantes-Coste et al. [9], who observed that nearly all patients (96.4%) attained spectacle independence after implantation of the Liberty 677MY IOL. To put these findings into perspective with other trifocal IOLs, Serdiuk et al. [15] reported that approximately two-thirds of patients implanted with the AT LISA tri achieved complete spectacle independence, whereas approximately 57% of those receiving the PanOptix IOL were able to dispense with glasses. However, these comparisons should be interpreted cautiously given the limited sample size of these studies and other potential methodological disparities when assessing spectacle dependence, residual refraction, and patient expectations, among other factors. Notably, these sources of variability were minimized in our comparative analyses (677MY vs. 677CMY), as the two cohorts were treated and measured in the same way.

Considering patient satisfaction, no meaningful differences were observed between the two IOLs. Patient satisfaction was elevated across all visual ranges for both cohorts, with more than 70% of patients reporting being “fully or very satisfied”. Similar overall satisfaction has been reported for other trifocal IOLs, such as the AT LISA trifocal diffractive IOL, for which 78.1% of patients reported satisfaction with their postoperative spectacle-free vision after implantation [21].

Interestingly, although most patients with both Liberty lenses were highly satisfied, the 677MY cohort showed a higher proportion of patients reporting complete satisfaction across most daily activities than the 677CMY cohort. However, given the exploratory nature of these analyses, these findings should be considered descriptive rather than confirmatory, particularly as no statistically significant between-group differences were observed. Consistent with this, no clinically or statistically significant differences were observed in the corresponding domains of the validated Catquest-9SF questionnaire between the two cohorts. Notably, although the 677CMY IOL showed more favorable intermediate VA across several objective measures, patient-reported satisfaction did not show a parallel pattern. This apparent discrepancy may reflect the multidimensional nature of patient satisfaction, which is influenced not only by VA but also by individual expectations, adaptation to the postoperative visual experience, and subjective perception of visual quality during daily activities, among others. Importantly, although numerical differences in the indication for surgery (cataract or CLE) and cataract maturity were observed between cohorts, these variables did not differ statistically significantly and are therefore unlikely to account for the observed distribution of satisfaction responses. Overall, these findings indicate that objective visual performance and subjective satisfaction may capture related but distinct dimensions of the postoperative visual experience.

Finally, patients reported on photic phenomena, which are generally expected to occur to some degree with any multifocal IOL [22]. Although a numerically higher proportion of patients in the 677CMY cohort reported experiencing halos, most symptoms were graded as mild (scores 1–2), and bothersome dysphotopsias remained uncommon in both groups. Accordingly, statistical comparisons did not reveal significant intergroup differences in either the frequency or perceived disturbance associated with halos, glare, or starbursts. These findings suggest that the optimized 677CMY design might not substantially alter the overall photic profile relative to the original 677MY.

Taking into consideration the safety profiles, no intraoperative adverse events occurred in either cohort, despite the advantage of the preloaded 677CMY lens, which requires less manipulation. No serious device-related postoperative complications have been reported for either model in the available literature [14]. Consistent with this favorable safety profile, CME and LASIK enhancement were infrequent in both cohorts. Although Nd:YAG capsulotomy rates were more frequent in the 677MY cohort than in the 677CMY cohort, this difference was not statistically significant after accounting for inter-eye correlation.

The main limitations of our study are its retrospective design, and the sequential rather than contemporaneous recruitment of the two cohorts, reflecting the clinical transition from the previous- to the next-generation IOL. As a consequence, temporal confounding from unmeasured changes over time cannot be completely excluded. Nevertheless, the consistency of the surgical team, operative technique, IOL power calculation, and refractive target across both cohorts reduces the likelihood that differences in these factors contributed to the observed outcomes. The sample size was also relatively limited and asymmetric between cohorts; however, both cohorts met the patient-level sample size requirement for the comparison of postoperative binocular intermediate VA. Nevertheless, the limited and unbalanced sample size may have limited the precision and statistical power for secondary and exploratory outcomes, which should therefore be interpreted with caution and further evaluated in larger, prospective studies. Additionally, this study included both eyes when analyzing monocular vision, as each eye undergoes an independent surgical procedure and provides clinically meaningful information; however, the correlation between fellow eyes within the same patient was accounted for in the statistical analyses. An additional limitation is the absence of an objective evaluation of the implanted IOL centration, visual quality, contrast sensitivity, or IOL-associated optical aberrations. Nevertheless, little or no difference would be expected between the two lenses in these aspects, as both share a similar underlying architecture. Finally, another limitation is the use of a non-validated questionnaire for patient-reported outcomes, which was previously applied successfully in other studies [17,23]. Nonetheless, we combined it with the validated Catquest-9SF questionnaire in order to strengthen the consistency of subjective patient reports.

## 5. Conclusions

Our findings suggest that the optimization of the Liberty IOL may provide a potential functional benefit, particularly for intermediate vision, while maintaining a comparable safety profile. Other clinical outcomes such as refractive predictability, dysphotopsic profile, and patient satisfaction appeared similar between the two IOL generations. These findings support the value of systematically comparing successive generations of IOL models to identify potential improvements in visual performance and to inform evidence-based clinical decision-making.

## Figures and Tables

**Table 1:** Preoperative demographic and clinical data.

	677MY Cohort (*n* = 13 Patients)	677CMY Cohort (*n* = 29 Patients)	Effect Estimate (95% CI)	*p*-Value
Patient-level variables				
Age, y	64.6 ± 9.0	64.4 ± 5.3	EMD −0.2 (−6.9 to 6.5)	0.944
Men/women, *n*	6/7	8/21	OR 0.4 (0.1 to 1.7)	0.298
Eye-level variables				
Indication (cataract/CLE), *n*	10/16	36/22	OR 2.6 (0.7 to 10.0)	0.161
Cataract maturity, grade	3.0 (3–4)	2 (2–3)	OR 0.4 (0.07 to 2.4)	0.309
IOP, mmHg	17.3 ± 4.9	15.6 ± 3.4	EMD −1.7 (−3.5 to 0.2)	0.076
Axial length, mm	23.0 ± 0.9	23.1 ± 1.0	EMD 0.1 (−0.3 to 0.6)	0.524
Keratometry, D	43.6 ± 1.1	43.6 ± 1.7	EMD 0.06 (−0.6 to 0.8)	0.850
Spherical equivalent, D	1.0 ± 2.8	1.3 ± 4.1	EMD 0.3 (−1.5 to 2.1)	0.931

Values are presented as mean ± SD and median (IQR). Effect estimates are expressed as estimated mean differences (EMD) for continuous variables and odds ratios (OR) for binary and ordinal variables, with 95% confidence intervals (CI). Patient-level variables were compared using the independent-samples Student’s *t*-test for the continuous variable and Fisher’s exact test for the binary variable. Eye-level continuous variables were analyzed using linear mixed-effects models and categorical variables using generalized estimating equations (GEE). The 677MY cohort was used as the reference group. CI: confidence interval; CLE: clear lens extraction; EMD: estimated mean difference; IOP: intraocular pressure; OR: odds ratio.

**Table 2:** Postoperative complications.

	677MY Cohort (*n* = 24 Eyes)	677CMY Cohort (*n* = 52 Eyes)	OR (95% CI)	*p*-Value
Any complication, *n* (%)	10 (41.7%)	14 (26.9%)	0.5 (0.1 to 2.1)	0.358
Posterior capsule opacification, *n* (%)	10 (41.7%)	10 (19.2%)	0.3 (0.1 to 1.4)	0.142
Cystoid macular edema, *n* (%)	0	3 (5.8%)		─
Refractive enhancement, *n* (%)	0	2 (3.8%)		─

Values are presented as *n* (%). The data were analyzed using generalized estimating equations (GEE).

## Data Availability

The data underlying this article cannot be shared publicly due to patient privacy and ethical considerations. The anonymized dataset supporting the findings of this study may be made available by the corresponding author upon reasonable request.
